# Text mining of hypertension researches in the west Asia region: a 12-year trend analysis

**DOI:** 10.1080/0886022X.2024.2337285

**Published:** 2024-04-14

**Authors:** Mohammad Rezapour, Mohsen Yazdinejad, Faezeh Rajabi Kouchi, Masoomeh Habibi Baghi, Zahra Khorrami, Morteza Khavanin Zadeh, Elmira Pourbaghi, Hassan Rezapour

**Affiliations:** a Faculty Member of the Iranian Ministry of Science, Research and Technology, Tehran, Iran; b Artificial Intelligence, University of Isfahan, Isfahan, Iran; c Department of Computer Engineering, Central Tehran Branch, Islamic Azad University, Tehran, Iran; d Department of Educational Science, Shahid Beheshti University, Tehran, Iran; e Ophthalmic Epidemiology Research Center, Research Institute for Ophthalmology and Vision Science, Shahid Beheshti University of Medical Sciences, Tehran, Iran; f Hasheminejad Kidney Center, School of Medicine, Iran University of Medical Sciences, Tehran, Iran; g Faculty of Advanced Sciences and Technology, Tehran Medical Sciences, Islamic Azad University, Tehran, Iran; h Department of Transportation and Urban Infrastructure Studies, Morgan State University, Baltimore, MD, USA

**Keywords:** Chronic kidney disease, hypertension, artificial intelligence, text mining, trend detection

## Abstract

More than half of the world population lives in Asia and hypertension (HTN) is the most prevalent risk factor found in Asia. There are numerous articles published about HTN in Eastern Mediterranean Region (EMRO) and artificial intelligence (AI) methods can analyze articles and extract top trends in each country. Present analysis uses Latent Dirichlet allocation (LDA) as an algorithm of topic modeling (TM) in text mining, to obtain subjective topic-word distribution from the 2790 studies over the EMRO. The period of checked studied is last 12 years and results of LDA analyses show that HTN researches published in EMRO discuss on changes in BP and the factors affecting it. Among the countries in the region, most of these articles are related to I.R Iran and Egypt, which have an increasing trend from 2017 to 2018 and reached the highest level in 2021. Meanwhile, Iraq and Lebanon have been conducting research since 2010. The EMRO word cloud illustrates ‘BMI’, ‘mortality’, ‘age’, and ‘meal’, which represent important indicators, dangerous outcomes of high BP, and gender of HTN patients in EMRO, respectively.

## 1. Introduction

Middle-income countries (MICs) face a disproportionately high burden of hypertension (HTN), as well as considerable population aging. It is therefore critical to understand how individuals move through HTN care stages. In a recent study, Mauer and colleagues longitudinally assessed how individuals with HTN move through care by using waves of cohort data from China, Indonesia, Mexico, and South Africa [1]. Their findings highlight the challenges faced by MICs in trying to improve HTN control and suggest that different approaches, beyond improving diagnosis, and initiating treatment, are needed.

The applying of computational artificial intelligence (AI) technologies for diagnosing disease outbreak trends and related studies have gained traction, given the potential to reduce cost and time necessary for extract hot topics and analyze them. Specifically, text mining is emerging as a viable method, leveraging available Latent Dirichlet allocation (LDA) and combining those with in visualizing methodologies of machine learning, topic modeling (TM), and clinical text mining to detect novel trends researches.

More than half of the world population lives in Asia [2,3] and HTN is the most prevalent risk factor found in Asia; Moreover, the most important reason of non-communicable diseases, such as increased stroke as the primary cause of disability and vascular death worldwide is HTN [4]. Indeed, high blood pressure (BP) levels increases linearly risk of recurrent ischemic and hemorrhagic strokes [5–7]. A recent meta-analysis showed that globally, stroke is the second-leading Level 3 cause of death (11·6% [10·8–12·2] of total deaths) in the world after ischemic heart disease (16·2% [15·0–16·9]) [8].

Stroke impacts of decreasing quality of life (QoL) and its higher average mortality rate compared to Europe, America, and Australia make it a serious problem in Asia. [2,3]. In 2019, age-standardized HTN prevalence was lowest in Canada and Peru for both men and women; in Taiwan, South Korea, Japan, and some countries in western Europe including Switzerland, Spain, and the UK for women; and in several low-income and MICs such as Eritrea, Bangladesh, Ethiopia, and Solomon Islands for men. HTN prevalence surpassed 50% for women in two countries and men in nine countries, in central and Eastern Europe, central Asia, Oceania, and Latin America. Globally, 59% (55–62) of women and 49% (46–52) of men with HTN reported a previous diagnosis of HTN in 2019, and 47% (43–51) of women and 38% (35–41) of men were treated. Control rates among people with HTN in 2019 were 23% (20–27) for women and 18% (16–21) for men. In 2019, treatment and control rates were highest in South Korea, Canada, and Iceland (treatment >70%; control >50%), followed by the USA, Costa Rica, Germany, Portugal, and Taiwan. Treatment rates were less than 25% for women and less than 20% for men in Nepal, Indonesia, and some countries in sub-Saharan Africa and Oceania. Control rates were below 10% for women and men in these countries and for men in some countries in North Africa, central and south Asia, and eastern Europe. Treatment and control rates have improved in most countries since 1990, but we found little change in most countries in sub-Saharan Africa and Oceania. Improvements were largest in high-income countries, central Europe, and some upper-middle-income and recently high-income countries including Costa Rica, Taiwan, Kazakhstan, South Africa, Brazil, Chile, Turkey, and Iran [9].

There are numerous articles published on the effects of HTN in Eastern Mediterranean Region (EMRO). To comprehensively analyze and extract the data of published documents using methods, such as review articles is difficult as well as time-consuming. Therefore, AI methods to analyze and extract the data effectively and comprehensively are needed.

To put it more clearly, while reading this large number of articles by traditional and non-robotic methods and by one person is very time-consuming, AI methods can speed up and reveal the knowledge published by the researchers of each country to the analysts. As an intelligent method in the process of health and disease risk factors, data mining has become very pervasive [10–21] and even with data mining, possible adverse effects can be achieved, including the positive effect found in one of the studies on vascular surgery in patients with HTN and published by Nature [22].

Given the similarity with data mining and clustering, it is no surprise that the use of topic models comes with a number of challenges typical for the application of unsupervised learning methods [23]. So in the analytical encounter with textual data – such as articles – text mining as a branch of data mining can be helpful; And more specifically, TM is a useful method of text mining technique that has been recently used widely in research fields [24]. TM is a kind of method that discovers hidden semantic structure from text corpus. It assumes that a document is a mixture distribution of topics, where the topic is a multinomial distribution about vocabulary [25]. It is based on text mining to retrieve information and identify latent topics in a collection of publications. The goal of TM is to reveal and better understand the construct of a phenomenon through the written text. Therefore, using TM, the aim of this study was to identify the multiple topics over the relevant documents on the HTN researches in EMRO.

## 2. Materials and methods

**Figure 1.**
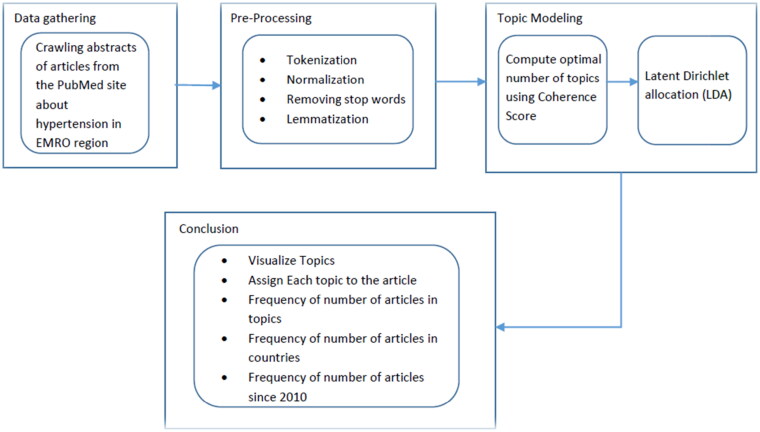
Detail of stages in this study from dataset acquisition to TM.

**Figure 2.**
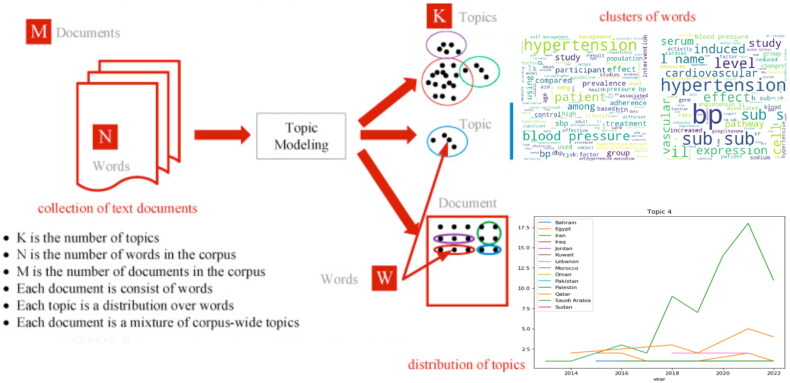
The infographic of input/output of the present analysis.

In this study, the overall proposed scheme can be divided into three sub-processes: (i) content crawling, (ii) pre-processing data, and (iii) TM deployment. Each step and their methods are explained in detail in the following sections. Figure 1 illustrates a flowchart of the dataset acquisition and analysis methodology and Figure 2 shows a big picture of the processes from the beginning to the achievement of the TM outputs:

### 2.1. Crawling contents

**Figure 3.**
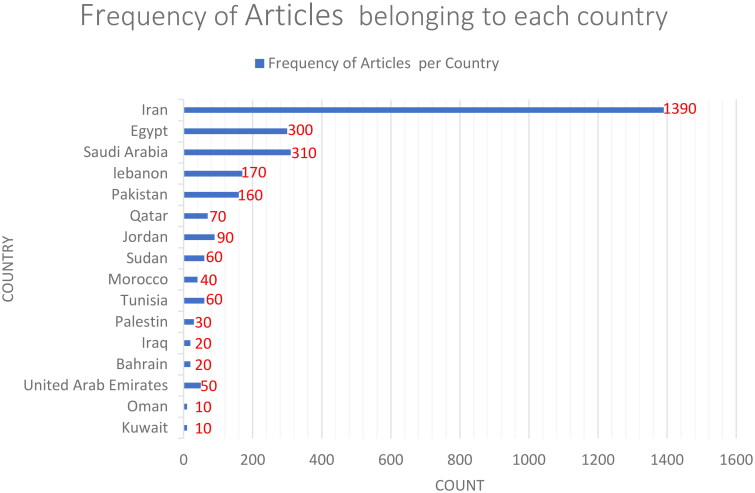
The frequency of articles belonging to each country of the EMRO.

In this phrase, web scrapping techniques and the Bio package in Python were used to gather data. In this study, 2790 articles on HTN, belonging to the countries listed in Figure 3, were gathered from the PubMed database. The selection and collection of articles were based on the relevance of their content to the concepts related to ‘hypertension’; The introduction of these concepts to the software robot was done based on terms suggested by half of the authors of this article, who are either doctors or have a specialized doctorate in the field of health. In order to detect and collect articles from those countries, the affiliation of the corresponding author institutions was considered. Figure 3 indicates the frequency of articles belonging to each country of the EMRO.

### 2.2. Data pre-processing

Regular expression was used in order to detect text patterns. After recognizing all URLs and numbers, they were replaced with URLs and #. In the second step, all words were converted to lower-case letters, and then punctuation marks were removed. In the next step, the stop words were removed, which included 179 common English stop words. Moreover, 590 words that did not have a special meaning in this analysis, such as ‘account’, ‘world’, ‘clinical’, and ‘first’ were removed as advised by the medical staff. Deciding which of the words have the least connection with the subject under study was also determined by the doctors and researchers in the field of health who are among the authors of this article.

In this study, the *n*-gram language model was implemented by using the Gensim library. *n*-gram language models are now widely used in text mining and natural language processing tasks. An *n*-gram model is a type of probabilistic language model for predicting the next item in a sequence of text or speech in the form of a (*n* − 1)-order. Having a good *n*-gram language model leads to predicting p (w | h) – the likelihood of seeing the word w given a history of previous words h – where the history contains *n* − 1 words. An *n*-gram of size 1 is referred to as a ‘unigram’; size 2 is a ‘bigram’; and size 3 is a ‘trigram’. When *N* is more than 3, this is commonly referred to as ‘four grams’ or ‘five grams’ and so on. Lemmatization was also developed by the Gensim library. By using a vocabulary and analyzing words morphologically, lemmatization groups together the inflected forms of a word and returns the base or dictionary forms of a word based on its intended meaning, identified as the lemma, or dictionary form. Lemmatization only keeps nouns, adjectives, verbs, and adverbs.

### 2.3. Topic modeling

The widespread use of TM for text mining provides a computational technique for realizing topics that obtain meaningful structures among documentary sources [26]. In machine learning and natural language processing, a topic model is a type of statistical model used to discover abstract ‘topics’ that occur in a set of documentary sources. TM is commonly used as a text-mining tool to discover hidden semantic structures in a textual context. TM procedures create ‘topics’, which are groups of analogous words [27]. This comprehension is captured by a topic model in a mathematical context, making it possible to examine a series of documentary sources and to discover the probable nature of the topics and the concept of the balance of topics in every document based on the statistics of the words in individual documents [27].

Furthermore, topic models are referred to as probabilistic topic models, which refer to statistical algorithms used to discover the hidden semantic structures of a large textual context [28]. The use of topic models makes it possible to organize and offer visions for users to understand large clusters of unstructured text-based contexts. Topic models were originally developed as a text-mining tool, but they are now used to detect instructional structures in data, such as genetic information, images, and networks. Moreover, their applications are expanded to other areas, such as bioinformatics and computer vision.

#### 2.3.1. Latent Dirichlet allocation (LDA)

LDA is a famous TM procedure for extracting topics from a specified corpus. LDA models describe the arrangement of words that are repeated together, occur frequently, and resemble one another. The Bayesian method is used in this probabilistic procedure. The model uses the examined documents and words to deduce the hidden topic structure, generating per-document topic distributions, P (topic | document), and per-topic word distributions, P (word | topic) [27].

Despite the fact that it was introduced by the perplexity-based technique, it is unlikely to result in distinct interpretations. LDA performs two functions: it finds topics in the corpus and assigns these topics to documents in the same corpus at the same time. Following preprocessing on the article abstracts, TM can be processed using the LDA algorithm, which is implemented in the Python Genism library. To answer the question ‘How does LDA work and how does it derive the specific distributions?’ we will go over its steps in general. All corpora, in the form of gathered documentary sources, can be considered as a document-word (or document term matrix) (DTM). The initial stage with textual data is cleaning, preprocessing, and tokenizing the text into words. The document-word matrix is broken into two matrices by LDA: DTM and Topic Word Matrix (TWM).

- There are probable topics (denoted by K above) in the DTM in advance, which may be present in the documentary sources;
- There are words (or terms) in the TWM that may be present in those topics.

To achieve sparse topic and word distributions for more understandable topics, small values on the Dirichlet hyper-parameters, α (parameter of Dirichlet prior to the per-document topic distributions) and β (parameter of Dirichlet prior to the per-topic word distributions) were chosen equal to 0.1 and 0.01, respectively. Choosing an optimal number of topics is complicated [29].

**Figure 4.**
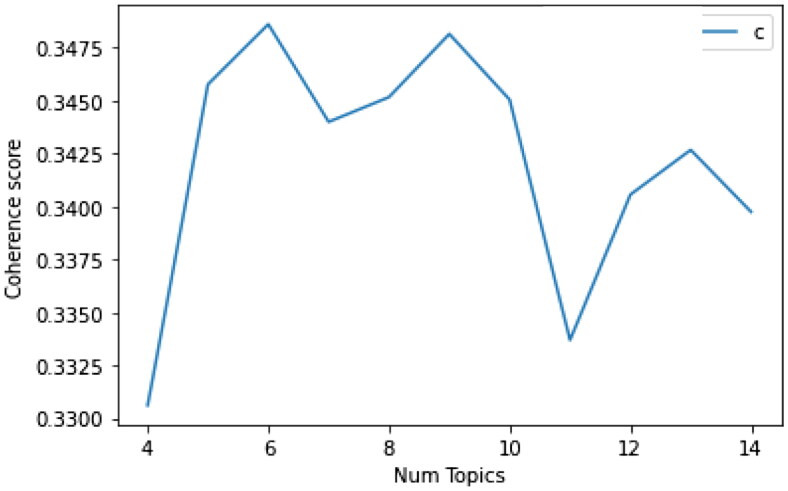
Coherence score of different topic numbers on LDA.

We need to build many LDA models with a different number of topics (*k*) and select the one that gives the highest coherence to find the best number of topics. Choosing a ‘k’ that represents the end of a dramatic growth in topic coherence usually yields meaningful and interpretable topics. Choosing an even higher value can sometimes result in more granular sub-topics. When the same keywords are repeated in multiple topics, it is likely that the ‘k’ is too large. Six topics were selected according to the calculation of the value coherence of 0.3486, and after running the LDA model with six candidate topics, each topic word and the frequency of each word were obtained. Figure 4 displays Coherence score of different topic numbers on LDA.

#### 2.3.2. Comparison of LDA with LSA, NMF, and BERTopic methods

In a nutshell, a topic model is a form of statistical modeling used in machine learning and NLP, as discussed earlier, that identifies hidden topical patterns within a collection of texts [30]. Those viewed as the most established, go-to techniques include LDA, latent semantic analysis (LSA), and probabilistic LSA [31]. More recently, however, newly developed algorithms such as non-negative matrix factorization (NMF), Corex, Top2Vec, and BERTopic have also received, and are continuing to attract, increasing attention from researchers [32,33].

Although such and LDA model, NMF also requires the data to be preprocessed, necessary steps to be performed beforehand include a classical NLP pipeline containing, amongst others, lowercasing, stopword removal, lemmatizing or stemming as well as punctuation, and number removal [34]. Additionally, for instance, while both models disclose users’ opinions on healthcare programs, but the LDA results appear to be more geographically oriented [35].

Lastly, when comparing BERTopic to NMF, a major shortcoming of NMF revolves around its low capability to identify embedded meanings within a corpus [36]. Considering that the algorithm depends primarily on the Frobenius norm [37], which is typically useful for numerical linear algebra, this issue ultimately leads to difficulties in interpreting findings [38]. Though NMF can effectively analyze noisy data [36], others argue that accuracy cannot be guaranteed [31].

## 3. Results

This section may be divided by subheadings. It should provide a concise and precise description of the experimental results, their interpretation, as well as the experimental conclusions that can be drawn.

### 3.1. Visualizing topic modeling with pyLDAvis

**Figure 5.**
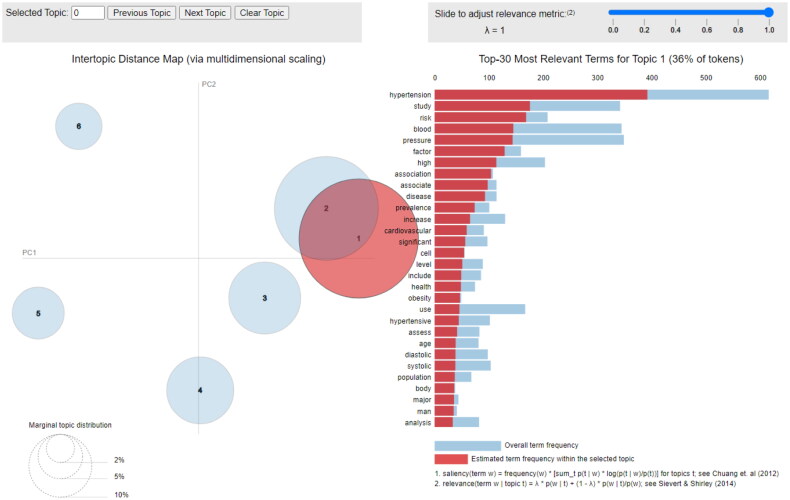
The six extracted ‘topics’, such as ‘clusters of words’.

pyLDAvis helps the elucidation of the topics in a topic model. Each circle represents a topic, and when the percentage of the total number of words in the corpus is greater, it results in the greater circle. The distance between the centers of the circles indicates the similarity between topics. The blue bars represent the total frequency of each word in the corpus. When no topics are selected, the blue bars of the words with the highest frequency will be displayed. Red bars represent the calculated number of times during which a given term was produced by a specified topic. The word ‘hypertension’ was the most frequently used in Topic 1, as shown in Figure 5.

#### 3.1.1. Naming the topics

A subject was chosen for each topic number for studying, analyzing, and presenting results more conceptually. The subject was selected based on the ten most likely words, the weight value of these words in the analysis, and studying the abstracts and keywords of the articles in each topic. Table 1 shows the frequency of words associated with each topic.

**Table 1.** Top frequented words associated with each topic.

Topics	Detailed content	Number of articles
Topic 0	Hypertension, patient, study, pressure, blood, use, control, HTN, management, treatment, medication, self, participant, hypertensive, and compare	82
Topic 1	Group, pressure, blood, effect, protein, systolic, diastolic, cholesterol, significant, High, sensitive, reactive, significantly, increase, and consumption	23
Topic 2	Study, effect, blood, pressure, sub, analysis, high, low, stress, patient, SBP, increase, compare, year, and mean	29
Topic 3	Sub, vascular, induce, system, renal, name, level, angiotensin, function, oxidative, mouse, effect, pathway, endothelial, and sodium	14
Topic 4	Hypertension, study, risk, blood, pressure, factor, high, association, associate, disease, prevalence, increase, cardiovascular, significant, and cell	121
Topic 5	Signal, model, feature, propose, system, cluster, ppg, center, use, achieve, clinical, blood, pressure, nox, and target	10

### 3.2. Identifying the semantic relationships between topics

**Figure 6.**
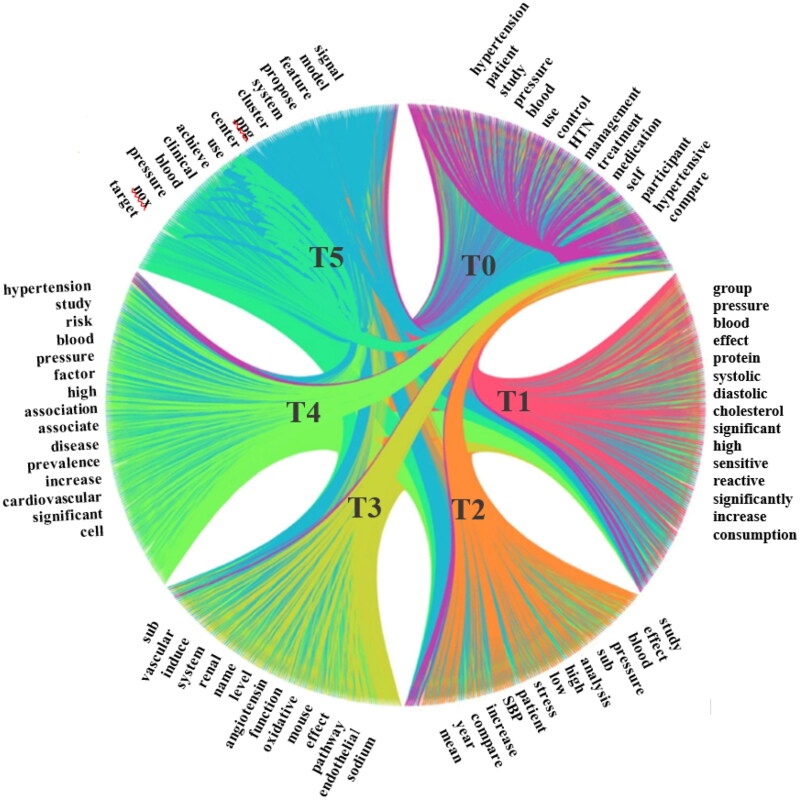
Identifying semantic relationship between 6 topics in Table 1 by co-occurrence map.

The semantic relationship between topics is identified, which are defined as co-occurrence statistics between topics, i.e., two topics are discussed in the same article; the way to identify such relationships is described as follows: 1) assigning each article to two topics with the highest probabilistic proportion in a topic proportion matrix; 2) measuring the relationships between two topics by using their co-occurrence statistics; and 3) generating a topic co-occurrence matrix and visualizing it [39]. As shown in Figure 6, each segment represents a topic, with the short label given in Table 1. The ribbons between segments stand for their semantic relationships, i.e., a stronger relationship between the two linked segments – such as T0 and T2, T0, and T3, T0, and T4 – is represented by a wider ribbon:

### 3.3. Content analysis of topics

The topics retrieved from LDA with k topics are named T0 − T (*k* − 1). In this survey, six topics were chosen by LDA, namely T0 − T5. Table 2 illustrates the selected subject as well as the 15 most likely words for each topic.

**Table 2.** The selected subjects in topics.

Topic number	Topic subject	The most 15 probable words in the topics
T0	Affecting Factors to the control or management of hypertension	Hypertension patient study pressure blood use control htn management treatment medication self participant hypertensive compare
T1	Variation in blood pressure and related factors	Group pressure blood effect protein systolic diastolic cholesterol significant high sensitive reactive significantly increase consumption
T2	Factors affecting hypertension	Study effect blood pressure sub analysis high low stress patient sbp increase compare year mean
T3	Hypertension and cardiovascular disease	Sub vascular induce system renal name level angiotensin function oxidative mouse effect pathway endothelial sodium
T4	Risk factors related to hypertension	Hypertension study risk blood pressure factor high association associate disease prevalence increase cardiovascular significant cell
T5	Controlling hypertension model/measuring techniques	Signal model feature propose system cluster ppg center use achieve clinical blood pressure nox target

#### 3.3.1. Distributions of articles in each topic

**Figure 7.**
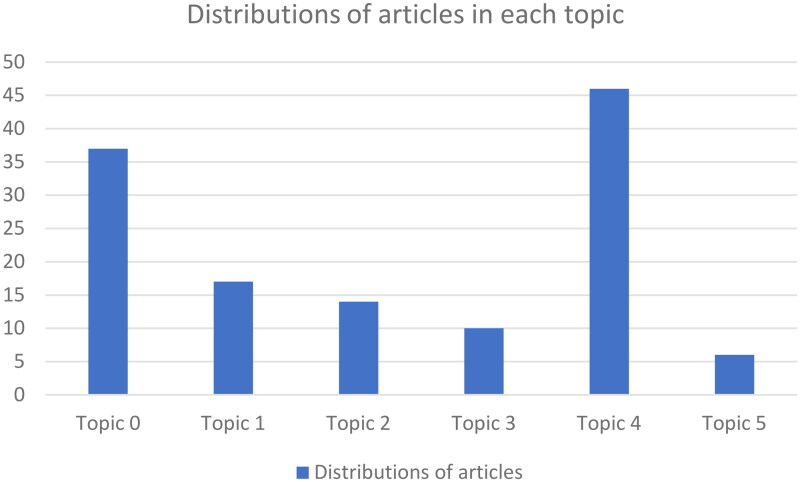
Distribution of articles in each topic.

Distributions of articles in each topic are shown in Figure 7.

**Figure 8.**
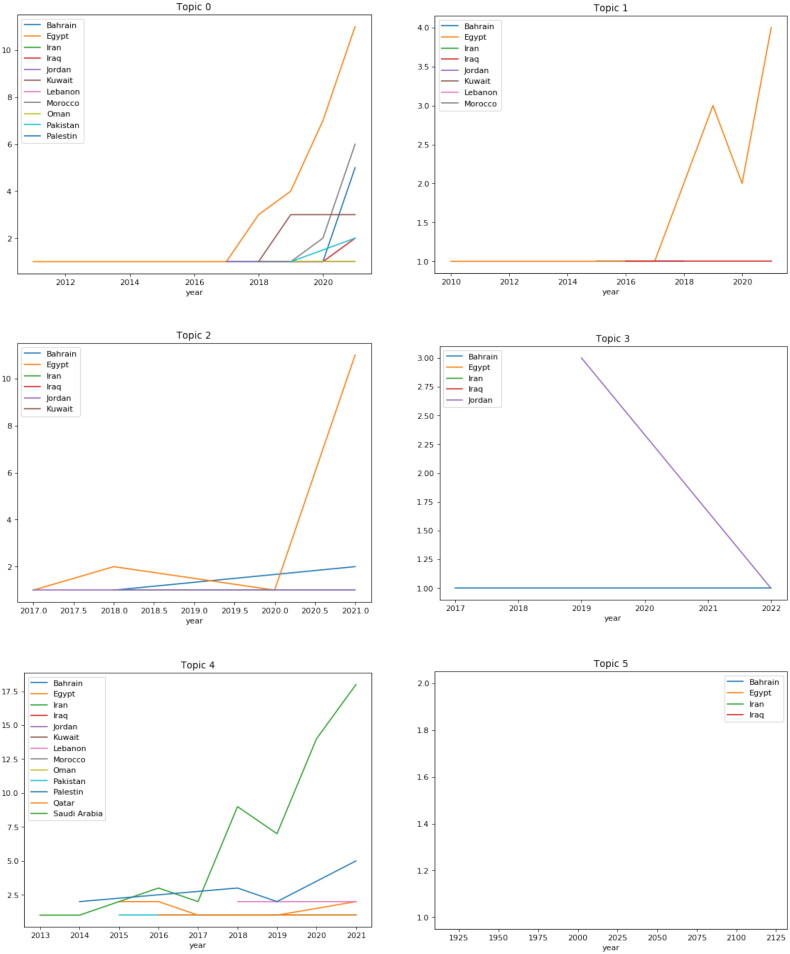
Distributions of articles ‘in each country on topics’ since 2010.

Distributions of articles in each country based on topics since 2010 are shown in Figure 8.

**Figure 9.**
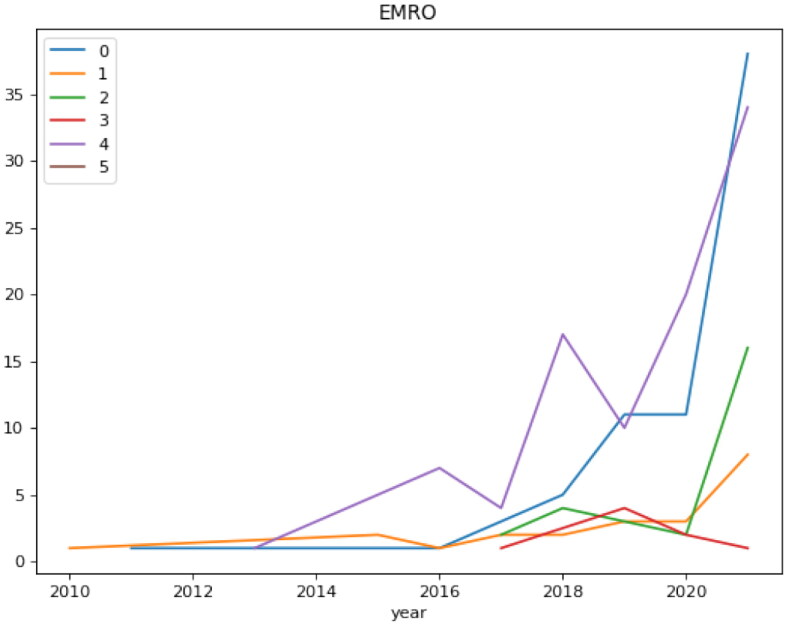
Distributions of articles based ‘on topics’ since 2010.

Distributions of articles based on topics since 2010 are shown in Figure 9.

#### 3.3.2. Word cloud of topics

**Figure 10.**
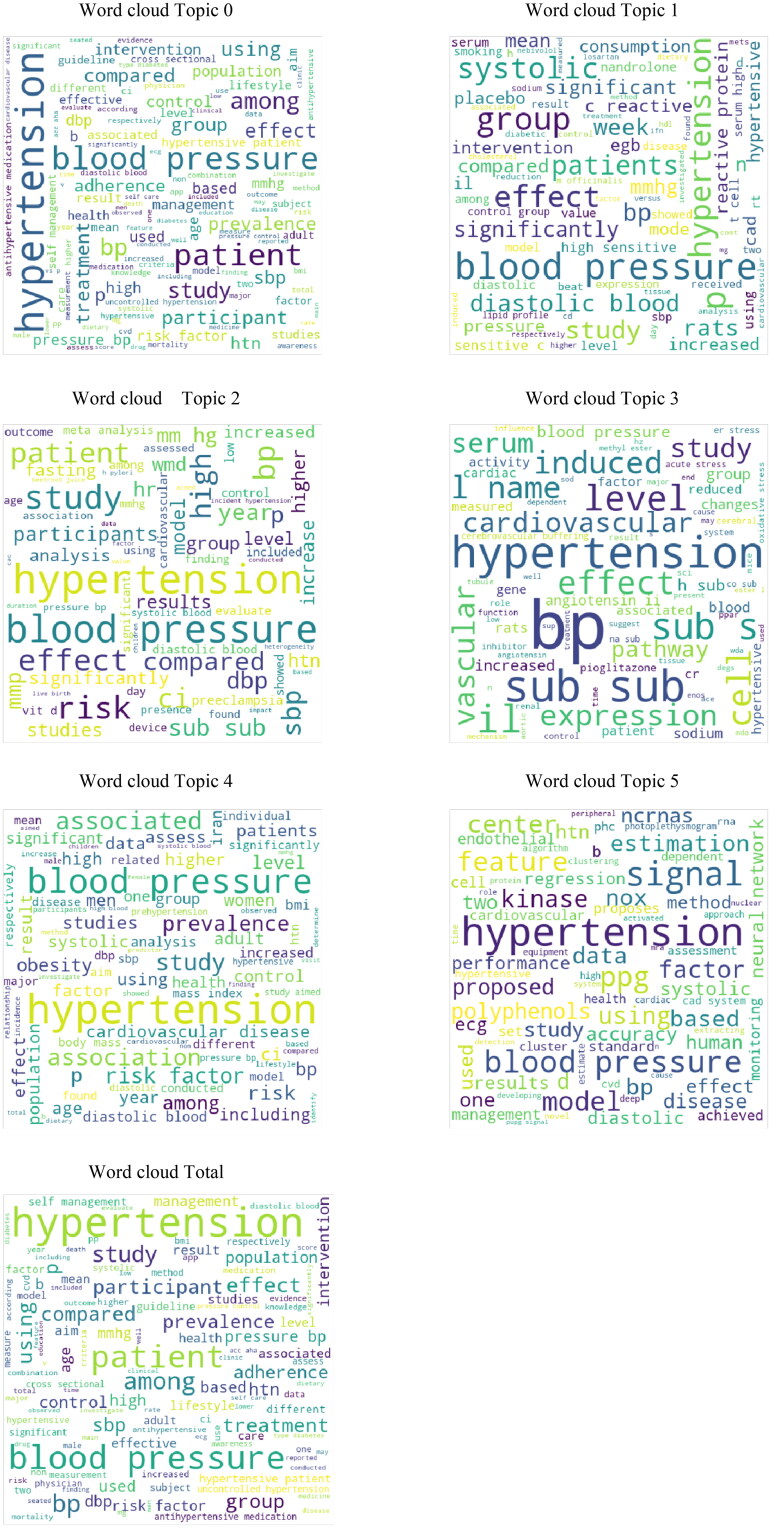
Word clouds of selected six topics and total words.

The word cloud is a data visualization technique, and the more a specific word appears in a source of text-based data, the bigger, and bolder it appears in the word cloud. It is possible to highlight important text-based data points by a word cloud, which has wide applications in data ­analysis. Figure 10 illustrates the word cloud of every topic.

It is noteworthy that big words in word clouds only mean frequent appearance, not importance or meaning; but the simultaneous appearance of some words in a super word is important and should be interpreted by the relevant expert. For example, in Figure 10, in ‘Word cloud Topic 3’ when we see the words ‘Vascular’ and ‘Shear Stress’ [40–44] together with BP (blood pressure [BP]), these need to be interpreted by vascular access surgeon; Or when ‘Cardiac disease’ and ‘kinase’ [45–47] are mentioned in ‘Word cloud Topic 5’ next to ‘Hypertension’, it is appropriate to bring the matter to the attention of a cardiologist.

#### 3.3.3. Distribution of topics per country

**Figure 11.**
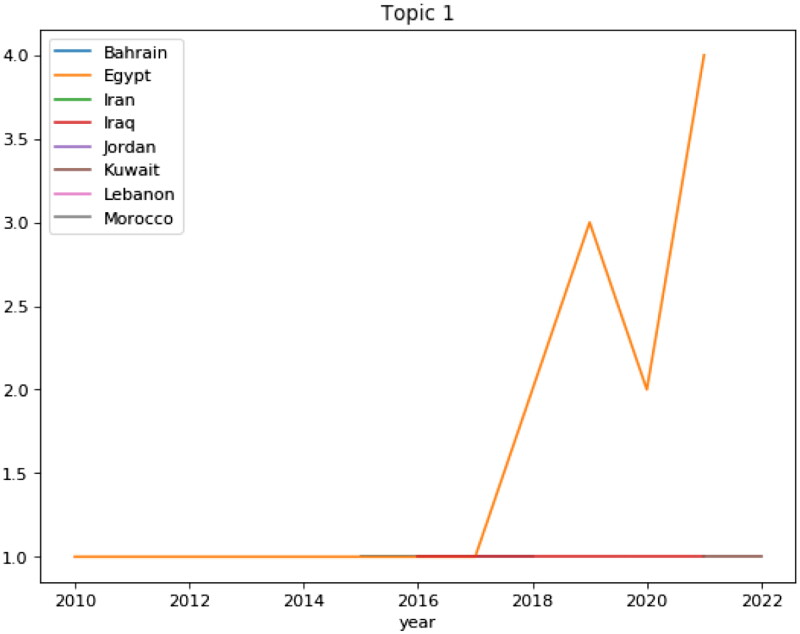
Distributions of articles based ‘on topic 1’ since 2010.

Figure 11 shows the published articles in the EMRO countries on changes in BP and the factors affecting it. Most articles in this field are related to Egypt, which has an increasing trend from 2017 to 2018 and its plummeted to the lowest point in 2020 and reached the highest level in 2021. Among the countries in the region, Iraq and Lebanon have been conducting research since 2010. This chart illustrated that Egypt is ahead of other countries in the region in terms of factors affecting changes in BP.

**Figure 12.**
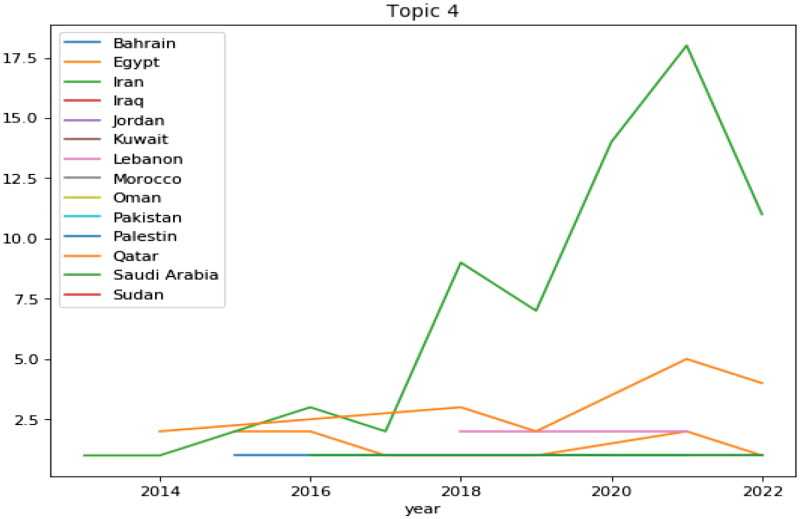
Distributions of articles based ‘on topic 4’ on the EMRO.

Studies on BP do not have the same level among EMRO countries, but differ significantly. As can be seen in Figure 12. In the period studied – i.e., the last 12 years – the Iranian studies started in 2013 and reached the highest level in 2021. After Iran, Egypt has the most research in this field. Lebanon and Pakistan, meanwhile, have published articles on the subject.

## 4. Discussion

HTN is a major risk factor for cardiovascular diseases and has a high prevalence in the EMRO [48]. The World Health Organization (WHO) reported that 30.7% of men and 29.1% of women in the EMRO were estimated to have HTN in 2008. HTN is a major risk factor for cardiovascular diseases, including coronary heart disease, heart failure, arrhythmia, and cardiomyopathy. There is also an increased risk of chronic kidney disease and stroke among hypertensive patients. According to The Global Burden of Disease Study, hypertensive heart disease accounted for 17.5 million disability-adjusted life years in 2015 [48].

Recently, an analysis of HTN in nationwide population is studied by cross sectional analysis method on the Korean adults aged 20 years or older [49].

Scholarly journals and data sources are increasingly available in electronic format making them more accessible to researchers and innovators. However, accessibility and availability do not mean that users can easily analyze the content and data that underpins scholarly output to find sought-after information or to develop new insights. Text mining comes with solutions for this problem offering automated methods to extract condensed information hidden within huge volumes of publications [50].

Text mining techniques, including LDA, LSA, and NMF, can be valuable tools for analyzing large volumes of textual data to identify patterns, topics, and trends within the literature. In the context of HTN research at EMRO, these methods can help researchers uncover hidden relationships, common themes, and emerging topics in published articles.

LDA is a probabilistic TM technique that can be used to identify clusters of words that frequently co-occur in documents. By applying LDA to a corpus of articles on HTN from EMRO, researchers can identify key topics and themes prevalent in the literature, such as treatment strategies, risk factors, comorbidities, or epidemiological trends.

LSA is another text-mining method that analyzes the relationships between terms and documents based on the underlying semantic structure of the text. By using LSA, researchers can uncover similarities and differences between articles, detect patterns in the use of terminology, and identify common concepts across the literature on HTN at EMRO.

NMF is a dimensionality reduction technique that can be applied to text data to extract latent features and patterns. In the context of EMRO’s HTN literature, NMF can help researchers identify clusters of related articles, extract important keywords or phrases, and reveal trends in research focus or methodologies over time.

### 4.1. Recommendations for future researches

By applying text mining methods to the corpus of published articles on HTN at EMRO, researchers can gain valuable insights into the evolving landscape of research in the region, identify emerging trends, and inform future research directions in the field.

Also, if geographical differentiation is included to compare research output based on the level of income (GDP per capita) in a country or to compare rural and urban areas, this can also help reveal research inequalities.

Moreover, temporal analysis can track if research on each topic or by specific countries is growing, declining, or plateauing over the 12-year span. This could inform future research investments and policy priorities.

Lastly, citation analysis of the articles could identify the most impactful or influential studies that may reveal pivotal developments like new *HTN* genes or breakthrough clinical trials. Highly cited works likely represent key research milestones.

## 5. Conclusions

TM as a branch of text mining used in this analysis and TM outputs describes the current situation of HTN researches published by EMRO authors and helps knowing notified patterns in recent articles, without necessity of reading all documents. In fact, the unsupervised TM method extracts topics and then show us the orientation of the edge of knowledge in this field and even inform non-specialists about different aspects of the field, without needing to have Prior Awareness or thought about different areas of that science or incorporated an idea into their research.

Note that this result may be obvious to BP specialists or cardiologists or neurologists, but we have obtained it with AI learning analyses. In addition, we did not work on a limited number of patients in a single country; instead, this study is an analysis of nearly 300 other studies over the entire EMRO. For example, by looking closely at the whole EMRO word clouds – located in the last word clouds of Figure 10 – even an engineer can learn that if researchers publish articles about HTN or BP subjects, they must be measured ‘sbp’ (systolic BP) as well as ‘dbp’ (diastolic BP). The mentioned word cloud (last of Figure 10) also contains words with special meanings on EMRO researches: for example, ‘BMI’, ‘mortality’, ‘age’, and ‘meal’, which represent important indicators, dangerous outcomes of high BP, and gender of HTN patients in EMRO, respectively.

## Institutional Review Board statement

Not applicable.

## Informed consent statement

Not applicable.

## Data Availability

All cleaned and pre-processed data and/or analyzed during this research are available from the corresponding author on reasonable request, but the original articles are available online in PubMed.
